# Improving management of patients with acute cough by C-reactive protein point of care testing and communication training (IMPAC^3^T): study protocol of a cluster randomised controlled trial

**DOI:** 10.1186/1471-2296-8-15

**Published:** 2007-03-29

**Authors:** Jochen WL Cals, Rogier M Hopstaken, Christopher C Butler, Kerenza Hood, Johan L Severens, Geert-Jan Dinant

**Affiliations:** 1 Maastricht University, Care and Public Health Research Institute, Department of General Practice, P.O. Box 616, 6200 MD Maastricht, The Netherlands; 2 Foundation of Primary Health Care Centres Eindhoven, Kloosterdreef 90, 5622 AB Eindhoven, The Netherlands; 3 Cardiff University, Department of Primary Care and Public Health, Neuadd Meirionnydd, Heath Park, Cardiff, UK; 4 South East Wales Trials Unit, Cardiff University, Neuadd Meirionnydd, Heath Park, Cardiff, UK; 5 University Hospital Maastricht, Department of Clinical Epidemiology and MTA, and Maastricht University, Care and Public Health Research Institute, Department of Health Organization Policy and Economics, P.O. Box 616, 6200 MD Maastricht, the Netherlands

## Abstract

**Background:**

Most antibiotic prescriptions for acute cough due to lower respiratory tract infections (LRTI) in primary care are not warranted. Diagnostic uncertainty and patient expectations and worries are major drivers of unnecessary antibiotic prescribing. A C-reactive protein (CRP) point of care test may help GPs to better guide antibiotic treatment by ruling out pneumonia in cases of low test results. Alternatively, enhanced communication skills training to help clinicians address patients' expectations and worries could lead to a decrease in antibiotic prescribing, without compromising clinical recovery, while enhancing patient enablement. The aim of this paper is to describe the design and methods of a study to assess two interventions for improving LRTI management in general practice.

**Methods/Design:**

This cluster randomised controlled, factorial trial will introduce two interventions in general practice; point of care CRP testing and enhanced communication skills training for LRTI. Twenty general practices with two participating GPs per practice will recruit 400 patients with LRTI during two winter periods. Patients will be followed up for at least 28 days. The primary outcome measure is the antibiotic prescribing rate. Secondary outcomes are clinical recovery, cost-effectiveness, use of other diagnostic tests and medical services (including reconsultation), and patient enablement.

**Discussion:**

This trial is the first cluster randomised trial to evaluate the influence of point of care CRP testing in the hands of the general practitioner and enhanced communication skills, on the management of LRTI in primary care. The pragmatic nature of the study, which leaves treatment decisions up to the responsible clinicians, will enhance the applicability and generalisability of findings. The factorial design will allow conclusion to be made about the value of CRP testing on its own, communication skills training on its own, and the two combined. Evaluating a biomedical and communication based intervention ('hard' and 'soft' technologies) together in this way makes this trial unique in its field.

## Background

This article describes the design of a cluster randomised clinical trial (RCT) evaluating the clinical efficacy and cost-effectiveness of a point of care C-reactive protein (CRP) test and enhanced communication skills in the management of lower respiratory tract infections (LRTI) in general practice.

About 80% of all antimicrobials are prescribed in primary care, and up to 80% of these are for respiratory tract indications, including acute cough. Respiratory tract infections are by far the most common cause of cough in primary care[1,2]. Broad spectrum antibiotics are often prescribed for cough, including acute bronchitis[3,4], and many of these prescriptions will benefit patients only marginally if at all (Number Needed to Treat = 17)[5], and may cause side effects (Number Needed to Harm = 33)[5]. Unnecessary prescribing, especially of broad spectrum antibiotics, drives antimicrobial resistance, wastes money and raises wrong expectations for patients. Microbial resistance topped the list of priority intervention needs in the World Health Organization report on Priority Medicines. Although the Netherlands is one of the lowest antibiotic prescribing countries worldwide, unnecessary prescribing for LRTI remains of great concern[6]. While unnecessary prescribing is a highly complex phenomenon, much of it relates to two factors, diagnostic uncertainty and patients' expectations. Interventions therefore should be aimed at clinicians to address these two factors. Previous studies with interventions aimed at general practitioners have resulted in less antibiotic prescribing[7,8].

### Diagnostic uncertainty and C-Reactive protein

LRTI represents a source of considerable diagnostic uncertainty for clinicians. Only a small number of patients with signs and symptoms of LRTI have pneumonia. Differentiating pneumonia from acute bronchitis on clinical grounds is often impossible and so clinicians frequently prescribe antibiotics empirically. However, as acute bronchitis is generally self-limiting, and benefits do not generally outweigh possible side effects from antibiotic treatment[5,9], rapid diagnostic tools to determine which patients will benefit from antibiotic treatment are urgently needed[10].

C-reactive protein is a marker of an acute inflammatory response. Several studies have indicated that CRP is probably the most feasible and accurate investigation to differentiate pneumonia from acute bronchitis in patients with LRTI in general practice [11-14]. We demonstrated in an earlier diagnostic study that for patients with a CRP less than 20 mg/l and who had no more than 1 of the 3 clinical predictors of pneumonia (dry cough, fever, diarrhoea), the absolute risk of pneumonia was reduced from 13% to less than 3%[11]. In daily practice a CRP value will only be useful when available during the initial consultation. CRP is available as a feasible point of care test for primary care and the test result can be available within three minutes. A low CRP test result is especially useful for reassuring both the doctor and the patient that further diagnostic testing and antibiotic treatment are not necessary. CRP tests can be done quickly, cheaply and with good reliability in everyday general practice using a finger prick blood sample[15]. In Scandinavia, point of care CRP testing is part of the routine evaluation of patients with LRTI in general practice, and its use has proved cost-effective[16,17]. In other countries including the Netherlands, however, GPs are still relatively unfamiliar with point of care CRP testing. The widespread introduction of point of care CRP testing in the Scandinavian countries was not accompanied by appropriate training for clinicians. This has led to an overuse of CRP testing in often mild respiratory tract infections. Slightly elevated CRP levels were too often followed by an unnecessary antibiotic course[18]. Therefore, thorough training in use and interpretation of test results is needed for a fair chance of success. This should include information about the diagnostic value of the test in addition to a focus on the fact that pneumonia can be excluded by a low test result.

### Patient expectations and enhanced communication within the consultation

Apart from diagnostic uncertainty, a range of other, 'non medical' influences come to bear on the decision to prescribe antibiotics[19]. Perceived patient pressure for an antibiotic, patient expectations and satisfaction are major factors influencing the decision whether to prescribe antibiotics [20-25]. Therefore, GPs need adequate communication skills to efficiently explain the pros and cons of antibiotic treatment, natural course and when to re-consult for this condition. Training GPs in enhanced consultations may therefore lead to easier and more acceptable non-antibiotic management[26]. Clinicians' communication skills in managing common infections are often sub-optimal[27,28]. In one of our studies, for example, carers' views/expectations on antibiotic treatment were elicited in only one of 39 audio taped consultations for children with respiratory tract infections[28]. Many GPs therefore do not routinely use optimal communication skills to efficiently implement a non-antibiotic management strategy that patients would find acceptable and is time efficient in time pressured consultations[29]. Although GPs perceive high patient expectations for antibiotics, evidence suggests that GPs are not good at estimating which patients expect antibiotics. Patient satisfaction is determined more by the quality of explanation and physical examination than whether or not they receive a prescription for an antibiotic[20,30]. Rollnick et al. developed an innovative communication skills training method which proved to be practical and acceptable to experienced clinicians[26]. With everyday clinical experience in the foreground and communication skills in the background this context-bound method proved successful in sore throat consultations and was well received by GPs[31]. We have shown that training clinicians using these methods was associated with changes in the behavior of clinicians and patients in consultations for common infections. In an interrupted time series pilot study, before training in our enhanced consultation skills, GPs in 10 consultations elicited patients concerns on two occasions and empathized three times in total[31]. No evidence was found of eliciting expectations of the consultation in general, or about antibiotics. After training, the same clinicians elicited patients concerns in 14 out of 15 consultations, elicited general expectations 8 times, elicited expectations about antibiotics in all 15 consultations, and empathized a total of 38 times. Before clinician training, patients expressed their expectations only once in 10 consultations and asked questions a total of 12 times. After clinician training, patients expressed expectations in 14 out of 15 consultations and asked questions a total of 33 times. Although the importance of studying an intervention like this in a large trial is obvious[32], as far as we know a full cost-effectiveness analysis has never been performed, to evaluate the efficacy of any intervention to improve health care providers' behaviour regarding antibiotics prescribing.

### Opportunities in LRTI

These studies on the potential for CRP and enhanced communication skills indicate that these interventions, either separately or combined, are able to have a positive effect on the prevention of misuse of antibiotics in LRTI. No point of care test has been evaluated together with context-bound training in enhanced communication skills.

Effective management of LRTI, we hypothesize, will involve a point of care test that performs well in primary care. It will also involve providing clinicians with the skills to efficiently and effectively communicate about the condition, its duration and implications to patients in a way that might make them accept a potential non-antibiotic management and facilitate patient enablement. However, some may argue that one of these interventions alone or indeed both together is required to enhance care. The factorial design of this study allows us to answer these questions.

### Research questions and outcome measures

This study will investigate the following research questions:

To what extent does the introduction of the C-Reactive Protein (CRP) point of care test and enhanced communication skills for managing LRTI in general practice, either separately or combined, have effect on:

#### Primary outcome measure

1. Immediate antibiotic prescribing rates

#### Secondary outcome measures

1. Clinical recovery and return to usual activities

2. Cost-effectiveness of point of care CRP and communication training

3. Patient enablement, satisfaction and future consulting intention

4. Diagnostic testing other than point of care CRP

5. Use of health services, including re-consultation

6. Subsequent antibiotic prescribing rates within 28 days of the initial consultation

### Ethical approval

The Ethics Committee of Catharina Hospital in Eindhoven, the Netherlands, approved the study protocol under number M05-1529, in compliance with the Helsinki Declaration.

## Methods/Design

This study is an open randomised controlled trial in general practice with randomisation at the practice level. The practices will be allocated to one of four groups (table 1):

**Table 1:** Intervention allocation

		**Communication skills training**	
		**+**	**-**
**C-reactive protein point of care test**	**+**	5 practices (10 GPs)	5 practices (10 GPs)
		100 patients	100 patients
	**-**	5 practices (10 GPs)	5 practices (10 GPs)
		100 patients	100 patients

1. Access to and training in point of care CRP

2. Context-bound training in enhanced communication skills for acute cough

3. Access to and training in the use of point of care CRP plus context-bound training in enhanced communication skills for acute cough

4. Usual care.

The GPs in groups 1 and 3 will be trained to use and interpret point of care CRP and those in 2 and 3 in enhanced communication with patients.

### Randomisation

A cluster randomised design has been chosen to avoid contamination. In order to allow assessment of the effects of each individual intervention and of combinations of interventions, compared with a control group, a factorial design for randomisation by practice will be used. Two randomisation procedures will be undertaken, one to create two groups for CRP/no CRP and a second for communication skills training/No communication skills training. The process will be stratified by the classification of the practices as above/below average for their ability to recruit patients (total number of practitioner clinics per practice per week). This will ensure a balance in recruitment potential between the two arms. Random permuted blocks of four will be generated for each randomization process. This will create two groups of 10 practices and balance at the margins for the stratification variable.

### Sample size calculation

In order to detect a reduction in antibiotic prescribing from 80% to 60%, with 80% power at a 5% significance level, an individually randomised study would require 175 patients. If we randomise 20 practices with an intra-cluster correlation coefficient (icc) of 0.06 this increases to 350 patients. In order to allow for a 90% follow-up rate for the primary outcome measure of antibiotic prescribing we intend to recruit 400 patients (20 per practice and 100 in each cell). We have previously demonstrated that 40 GPs can recruit over 400 patients suspected of having a LRTI in two winter seasons[11].

### General practices

A total of 20 general practices will be recruited, with a fixed number of two GPs per practice participating in the study. The practices are located in the South-eastern part of the province of Noord-Brabant in the Netherlands. This region compromises both urban and rural areas. Recruited practices will be geographically spread throughout this region.

### Patients

**Figure 1:**
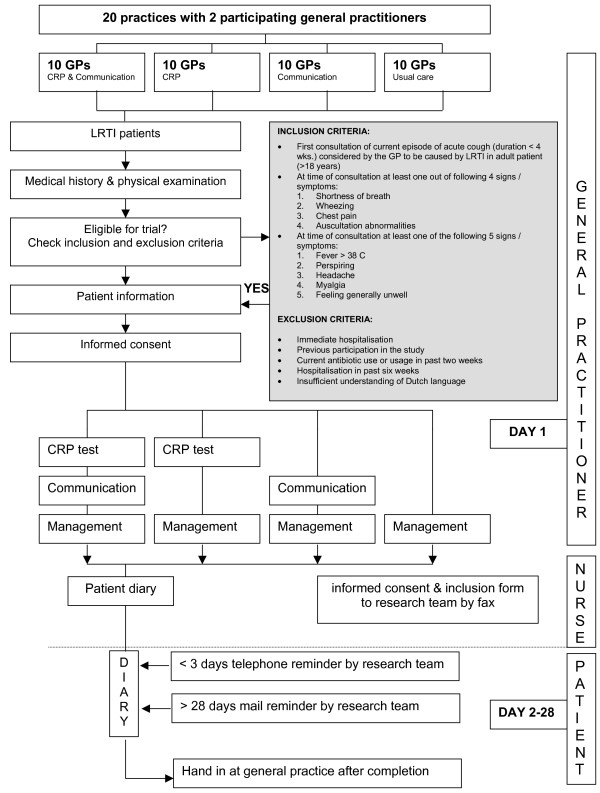
Study flow chart.

The GPs will approach sequential eligible adult patients during regular consultation hours. Patients are eligible if they present in general practice with an acute cough, lasting no more than 4 weeks, considered to be caused by LRTI according to the GP. In addition, the patient needs to have at least one focal and one systemic sign/symptom[33] (Figure 1). Children will not eligible, since the diagnostic value of CRP has not been investigated for LRTI in children, consultations with children and their parents will be different with respect to communication styles and strategies, and diagnostic and prognostic factors are not automatically applicable in infants. Exclusion criteria are described in figure 1.

### CRP point of care test

**Figure 2:**
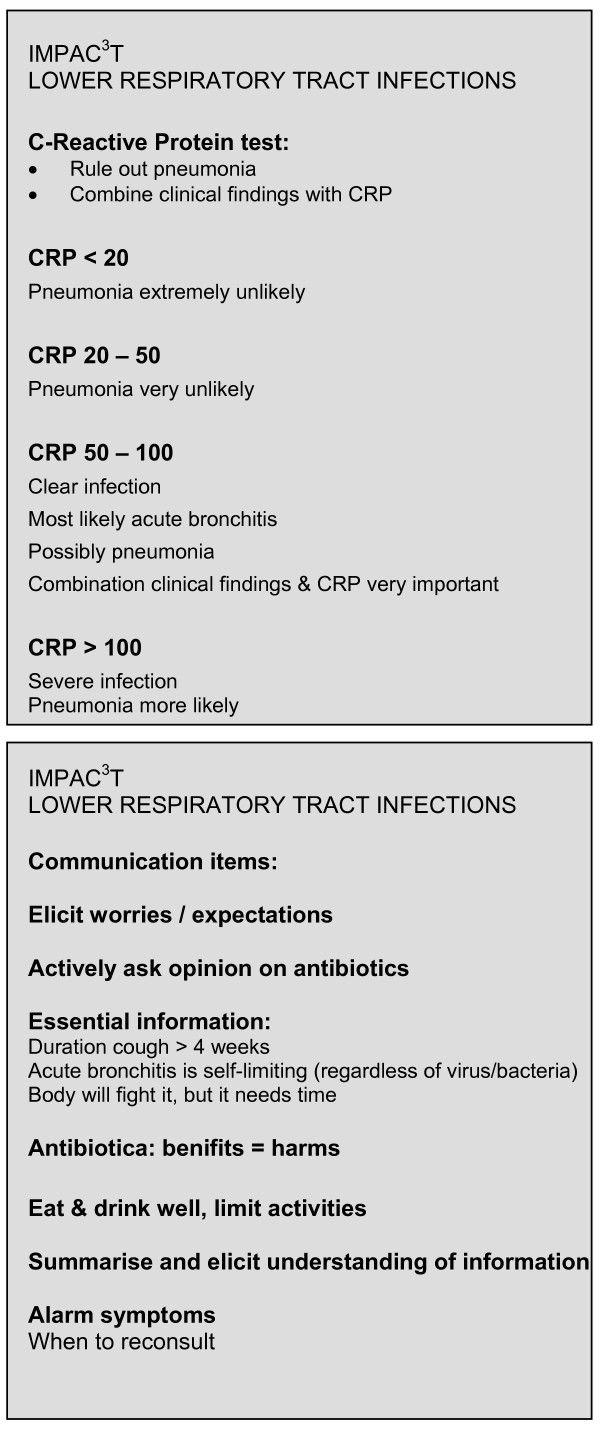
Desk reminders for GPs allocated to the CRP and/or communication skills interventions.

20 GPs will have a CRP test device in their practice. CRP analysis will be carried out during the consultation. The NycoCard^® ^II Reader, product of Axis-Shield, will be used, in accordance with the manufacturer's instructions. The test system is based on an immunometric principle and consists of 1) a liquid for sample dilution and lysis of cells, 2) a test card with CRP-specific monoclonal antibodies coated to a membrane, 3) a conjugate solution with monoclonal antibodies coupled to ultra small gold particles and 4) a washing solution. One single drop of blood from a finger prick is needed to perform the test. A capillary collects 5 μl blood which is diluted in the 0,4 ml dilution liquid. 50 μl of this diluted sample is applied to the test well of the device. When the sample flows through the antibody-coated membrane, C-reactive proteins are forced to react and bind to the antibodies on the membrane. CRP trapped on the membrane will bind the antibody gold conjugate. Unbound conjugate is removed by one drop of washing solution. In the presence of pathological levels of CRP in the sample, the membrane appears red-brown with color intensity proportionally to the CRP concentration. The color intensity is measured quantitatively with the color densitometer NycoCard^® ^Reader II. The measuring range for whole blood samples is 8 to 250 mg/l. In the measuring range the test response is directly proportional to the concentration of CRP in the sample. For internal quality a control positive will be measured to confirm the efficacy and correct performance of the test. The control liquid is supplied with every test kit and will be run after opening of a new kit, which is once in every 24 regular tests. CRP measurements with Nycocard^® ^have proven to be valid and robust in general practice[34]. The result will be available within three minutes. This means that GPs can use the test result within the consultation. The 20 GPs who are allocated to the CRP intervention will be given guidance about the interpretation of CRP results. This information will include a comprehensive summary of the literature on LRTI and the role of CRP. We will not instruct the GPs in a prescription style fashion. Focus will be on informing them on the additional diagnostic value of CRP in ruling out pneumonia. GPs will decide on management including possible antibiotic treatment in every patient. CRP may complement their clinical findings and help in deciding upon diagnosis and treatment. The desk reminder for CRP interpretation in LRTI can be seen in figure 2. Practice assistants will be trained to use the NycoCard^® ^II Reader by the Dutch distributor who has over 10 years of training experience. GPs and assistants will be able to consult a 24-hour helpdesk for technical assistance. Questions on interpretive issues can be submitted to the research team. For specific test handling questions, we will also create a direct telephone number connecting the assistant with a clinic where assistants have extensive experience with handling point of care CRP testing. Once the CRP devices have been installed and training sessions have been performed, practices will get a 8-week run-in period before inclusion starts for familiarisation with the device and interpretation of the results.

### Communication training

The communication training is based upon a patient-centred strategy to achieve shared decisions about investigation and treatment of acute infections. The intervention is based on the elicit-provide-elicit framework, which has its background in motivational interviewing and which has been refined for use in primary care[26]. The context-bound training for acquiring these communicating skills involves one two-hour training seminar at a central location, preceded and followed by consulting with simulated patients in routine surgeries. It involves the following specific elements:

#### 1. Simulated patient in clinical encounter, part 1

Two experienced simulated patients will be trained to simulate a patient with acute bronchitis persisting for two weeks. The patient is worried about the duration of the cough and has distinct sleeping problems and impaired daily activities due to the cough. The simulated patients will consult with participating GPs at their own surgeries during regular surgery hours. The simulated patients will carry an audiotape device to record the conversation, which will be transcribed by trained typists.

#### 2. Seminar on shared decision making

Within one week after their first simulated patient consultation, all 20 GPs will gather for a training seminar on enhanced communication skills. Three seminars will be held, with 5–8 GPs participating per session. All session will have the same format and content (see below). An experienced moderator from the Department of Vocational Training in General Practice will lead the seminar. The GPs will be encouraged to actively participate in all sections of the training. After a brief introduction, the GPs will receive transcripts of their first simulated patient contact. They will be asked to read their own transcript and make notes. Subsequently, the moderator will invite them to share their reactions to their transcripts and to identify noteworthy aspects. These can be of any kind; structure, vocabulary or occurring difficulties in management. The focus will be on the acute cough patient. This will be followed by a short presentation on current views and insights on LRTI. The contrast between evidence from research and figures from daily practice will be highlighted to stress the importance of enhancing LRTI consultations. GPs will be given the opportunity to contribute their own suggestions for enhancing LRTI consultations. The elicit-provide-elicit framework will be outlined[26], where the GP first *elicits *what the patient knows about his condition and what the patient's main worries and expectations are. Crucially, the GP actively asks how the patient feels about antibiotics. This serves two purposes; firstly, to provide the GP with the patient's expectation and hope for antimicrobial treatment (making patients' expectations explicit). Secondly, it enables the GP to openly discuss his opinion on antibiotic treatment, mentioning the balance of possible benefits and harms of antibiotic treatment. Secondly, the GP *provides *information relevant to the patients' individual understanding and interest. This includes findings from the medical history and physical examination. Then the GP *elicits *the patients' interpretation about what has been said and done, and discusses implications for help seeking behaviour. Specific alarm symptoms should be mentioned to provide exact information to the patient about when to re-consult. All communication is based on an assessment of patients' information needs, thus achieving efficiency by targeting information to individual needs. Practice-based examples will be presented in video clips. Finally, GPs will be asked to attempt to identify the specific aspects, which need most attention during their consultations. Supported by a desk reminder (Figure 2) we will ask the GPs to implement the specific steps of the communication training in their routine acute cough consultations.

#### 3. Simulated patient in clinical encounter, part 2

The GPs will get the opportunity to use the enhanced communication skills in daily practice during eight weeks before inclusion start. The two simulated patients will now switch practices, visiting the practices they did not visit, while playing a similar acute bronchitis role. Trained typists will once again transcribe the audio-recorded consultations. These transcripts will be copied and made anonymous, after which they are sent to a colleague participating in the study. This GP will be asked to write comments on the transcript based on the enhanced communication method and to add possible suggestions for improvement. The project team will edit or add comments if needed. Finally, the peer-reviewed transcript will be returned to the GP who conducted the consultation. Commenting on a colleague's transcript may promote GPs' critical reflection on their own peer-reviewed transcripts. Thus, participating GPs will benefit from reading and feeding on a colleague's transcript and from reflection on their own transcript.

### Data collection

Sequential elegible patients will be asked by their GP to participate. During this consultation, the GP will take written consent and note the inclusion criteria on the trial inclusion form. The practice nurse will give each participant a simple self-complete symptom diary after the consultation. After general questions on cough and the consultation, patients will be asked each day to complete a 6 item scale, with responses to statements ranging from 0 to 6. A total symptom score will be derived from this scale. Items relate to coughing, phlegm, shortness of breath, sleep disturbance, impairment of normal daily activities, and generally feeling unwell. This symptom diary has previously been used in a large LRTI study[35]. Patients will then be followed-up for 28 days, or if still unwell, until recovered with a maximum follow up of ten weeks. Furthermore, weekly questions will record time off work, medication use and re-consultation. The recruiting GP will fax the informed consent form and inclusion form to the research team in Maastricht on the day of inclusion. The clinical effectiveness analysis will be based on diary responses. A single Likert type scale response item will assess satisfaction with the consultation. Patient enablement will be measured using the Patient Enablement Instrument [36] translated into Dutch. Concurrent validation will be undertaken. In addition, the research team will retrieve data on diagnosis, antibiotic prescription, re-consultation, diagnostic testing other than CRP and related medical consumption from participating patients' electronic medical records over 28 days follow-up. A societal perspective will be used for the economic evaluation. Thus, for all four groups, in addition to costs to the health care system, we will take into account medical costs borne by patients and production costs due to illness related absence from work. Use of health care facilities will be prospectively recorded in a standardised way including all re-consultations, telephone consultations, out-of-hours consultations, and hospital admissions, (repeat) prescription of antibiotics (type, dosage, duration) and additional diagnostic tests, including bacteriological culture and X-ray. A patient diary will be the basis for assessing actual medication use, other medical costs to the patient, and absence from work[37].

The research team will retain transcripts of simulated patient consultations. Transcripts before the seminar will be compared with those afterwards for evidence of acquisition of the enhanced communication skills (competence).

### Patient follow-up

The respondent burden will be kept to a minimum. The research team will contact patients by telephone within four days to offer further information about study participation and to iron out eventual problems concerning study materials. Reminders to complete study documents will be sent by post to patients after 14 days, and patients will receive a reminder postcard at the end of the follow up period.

### Analysis

Statistical analyses will be performed with the aid of the SPSS and STATA statistical packages. The primary analysis will be intention to treat and will compare the marginal effects of the two interventions on antibiotic prescribing at the index consultation and incorporate an interaction effect. The interaction term will be included to test for a synergistic or antagonistic relationship between the two interventions. This will be tested using a three level logistic regression model to account for variation at the practice/GP/patient. If there is no indication of random effects at a GP level, then this will be reduced to a two level model with GP seen acting as a patient level covariate.

A four level linear regression model will be fitted to the symptom scores (logged) to account for practice, GP, patient and repeated assessments over time. The correlation between repeated assessments within individual patients will be modelled to allow for greater correlation between assessments, which are closer in time. The effect of interventions would be seen in the comparison of the slopes of symptom scores over time in the groups, i.e. having faster (or slower) recovery rates[38]. Sensitivity analyses on the symptom scores will be undertaken to assess the impact of loss to follow-up by either interpolating from available data using the estimated slope of symptoms from the model or using last value carried forward. The later is unlikely to arise in practice for an acute infection, however provides a useful extreme bound in which to interpret results.

Secondary outcomes (satisfaction, enablement, re-consultation and other investigations) will be compared between the two groups using three level regression models (linear and logistic where appropriate). No formal sub-group analyses are planned. However, exploratory analyses will investigate the influence of patient and practitioner demographics on outcomes. The statistical uncertainty regarding the cost-effectiveness of the different strategies will be assessed using non-parametric bootstrap resampling techniques and result in estimates of net monetary benefit values. For the groups of GPs trained in enhanced communication skills and/or CRP testing, the cost analysis will be extended beyond patient level data. Training will lead to additional costs (organising and giving the training and GPs' time investment) which will be included in the analysis[39]. Such overhead costs are basically one-time costs and can therefore be regarded as fixed costs that will be measured at the level of the trained GPs and attributed to an individual patient, which will be done on the basis of a division calculation as proposed by Mason et al (2001)[40]. The trial will be reported following CONSORT guidelines, extended for cluster randomised trials[41].

## Discussion

This trial will be the first randomised controlled trial to evaluate the use of point of care CRP testing and enhanced communication skills training, on the management of acute cough due to LRTI in primary care. Both are promising interventions in reducing unnecessary antibiotic prescribing. A systematic evaluation in daily general practice is required to assess effectiveness. Our inclusion criteria are quite broad as a reflection of daily practice. LRTI is such a commonly seen illness among the full range of patients in primary care that we think the inclusion criteria should enable us to investigate all these patients. Primary inclusion criterion is the GP's suspicion of LRTI being the primary cause of the acute cough episode. GPs are daily facing difficulty in deciding upon management for these patients. The pragmatic approach of this trial leaves them very close to daily practice when considering recruiting the patient for this study. We did however define a group of focal and a group of systemic signs and symptoms in the inclusion criteria, which indicate that this patient has signs and symptoms of an infection of the lower respiratory tract.

A low CRP test result, expected to occur in at least half of these patients with LRTI, will be reassuring for both the GP and the patient that the illness is mild and most probably self-limiting. On the other end of the spectrum, very high test results will help the GP in considering antibiotic treatment or referral to a hospital. To achieve this GPs must be aware of the usefulness of the test and they should be able to properly interpret results. These are crucial conditions for a positive effect of the CRP intervention. If GPs are not aware of the value of the test, other important influences such as time pressure and patients' expectations may dilute any possible effect on antibiotic prescribing. If GPs are able to build the communication skills, which take the latter influences into account, into their routine consultations, long term competence and performance have to be monitored, since maintenance of acquired communication skills is difficult. So even when GPs understand the theory of the test and the principle of the enhanced communication, the question remains whether they will use it to adjust the management of LRTI patients.

We chose a factorial design, as it will enable us to efficiently investigate the interventions by including all participants in both analyses. Furthermore it will give us the opportunity to consider both the individual effects of both interventions and the benefits of receiving both interventions together. Factorials trials do require special considerations at the analysis stage. Interaction between the interventions needs to be investigated, since this can have effect on the power of the study[42].

In this trial both interventions are targeted at the GPs with impact on patient outcomes. Randomisation at practice level has been chosen in this trial, because there is less risk of contamination by GPs concurrently managing usual care patients, while other GPs manage their patients under the experimental regimen in the same practice. However, since patients yet have to be recruited after randomisation post-randomisation recruitment bias deserves special attention. One may expect that practices not receiving a desired intervention may gradually loose enthusiasm to recruit. As a strategem to prevent this, those practices allocated to the usual care arm, as well as those allocated to one of the arms to which only one intervention will be allocated, will be informed that they will receive a CRP device and/or the communication training after the study period. Recruitment rates across the four groups have to be monitored and baseline characteristics will be described to ensure comparability. Because of the small number of practices involved, randomisation will be restricted carefully with regard to recruitment ability. With a small number of clusters and a large number of patients this trial may be difficult to analyse and risk of loss of power exists if one practice should drop out. In cluster randomised trials the outcome for each patient cannot be assumed to be independent of that of any other patient since the patients within one cluster (practice) are more likely to have similar outcomes. Therefore multi-level analysis will be performed.

Prediction of sample size in cluster randomised trials is difficult given the fact that not only the expected effect size has to be estimated, but also the anticipated cluster size and intra-cluster correlation coefficient[43]. Despite these limitations analysis on both cluster level as well as individual level will provide us the best information on the outcome of this study[44]. Results will be reported with full transparency with regard to the factorial and cluster design[41,42].

The study will allow us to get more insight into the natural course of this common but still under-researched illness. The long follow-up of 28 days and the daily patients' evaluation of number and extent of their symptoms will provide crucial information on natural course, burden and impact on daily activities.

To our knowledge, both from the point of view of interventions and study design, this is a unique study. It will be the first to evaluate complex interventions separately and combined, that focus on the biomedical evaluation of the illness and patient centered communication. The pragmatic nature of the study, which leaves treatment decisions up to the responsible clinicians, will enhance the applicability and generalisability of findings. If these interventions decrease antibiotic prescribing and enhance patient satisfaction with favourable clinical outcomes, they could be used to enhance the quality of a large number of consultations in primary care, both for common infections and for other conditions. A full cost-effectiveness analysis to evaluate the efficiency of the interventions is needed in this regard as well and will therefore be performed. This has never been done before for an intervention to improve health care providers' communication regarding antibiotics prescribing.

If these complex interventions are shown to be effective and cost-effective, they may be refined as a result of the research and we will promote uptake in everyday care.

## Abbreviations

GP General practitioner

LRTI Lower respiratory tract infection

CRP C-reactive protein

ICC Intra-cluster correlation coefficient

RCT Randomised controlled trial

## Competing interests

The author(s) declare that they have no competing interests.

## Authors' contributions

JC is the principal investigator and wrote the manuscript. RH contributed to writing of the manuscript, background and CRP intervention of the study protocol; supervisor of the project. CB contributed to writing of the manuscript, designed the study protocol, contributed to the background information and to the communication part of the study; supervisor of the project. KH is the trial statistician, contributed to the drafting of the study design; supervisor of the methodological and statistical data processing. JS contributed to the drafting of the study design and the health economics aspects of the study. GD is the overall project leader and grant applicator.

All authors have read and approved the final version of the manuscript

## Funding

This study is funded by the Netherlands Organisation for Health Research and Development (ZonMW, Doelmatigheidsonderzoek), grant number 945-04-010. Dr Hood is funded by the Wales Office for Research and Development in Health and Social Care. None of the sources of funding influenced either the study design, the writing of the manuscript or the decision to submit the manuscript for publication.

## Pre-publication history

The pre-publication history for this paper can be accessed here:


